# Evaluation of Broccoli Extract for Enhancing Primary Tooth Enamel Microhardness: An In Vitro Study

**DOI:** 10.1002/hsr2.70505

**Published:** 2025-02-19

**Authors:** Sadighe Mozaffar, Mehrdad Karimi, Ali Ismail, Morteza Banakar

**Affiliations:** ^1^ Department of Pediatric Dentistry, Faculty of Dentistry Shahed University Tehran Iran; ^2^ Department of Traditional Medicine Tehran University of Medical Sciences Tehran Iran; ^3^ Department of Prosthodontics, School of Dentistry Shahid Beheshti University of Medical Sciences Tehran Iran; ^4^ Centre of Molecular Medicine and Diagnostics (COMManD), Saveetha Dental College & Hospitals, Saveetha Institute of Medical and Technical Sciences Saveetha University Chennai India; ^5^ Dental Research Center, Dentistry Research Institute Tehran University of Medical Sciences Tehran Iran

**Keywords:** broccoli, caries prevention, enamel microhardness, fluoride, primary teeth

## Abstract

**Background and Aims:**

Dental caries remains highly prevalent among children. This study aimed to evaluate the efficacy of aqueous broccoli extract in enhancing the microhardness of demineralized primary tooth enamel compared to a standard fluoride treatment.

**Methods:**

An in vitro study was conducted using 30 extracted primary second molars, which were sectioned and polished. Baseline enamel microhardness was assessed using a Vickers hardness tester. Demineralization was induced using a cola drink (pH 4.5) for 8 min. The broccoli extract was prepared by air‐drying fresh florets, stems, and leaves at room temperature, followed by aqueous extraction with distilled water and filtration. The samples were randomly assigned to receive a 10‐min treatment with either aqueous broccoli extract (Group B) or 1.23% acidulated phosphate fluoride (APF) gel (Group F). Microhardness was measured posttreatment, and percentage changes between time points were compared using statistical analysis.

**Results:**

After demineralization, broccoli extract, and fluoride gel significantly improved enamel microhardness. The broccoli extract group exhibited a greater recovery in microhardness (+44.95% vs. +20.78%, *p* = 0.008) compared to the fluoride gel group. However, the overall reduction in microhardness from baseline to final measurement showed no statistically significant difference between the two groups (*p* = 0.077).

**Conclusion:**

Aqueous broccoli extract demonstrated comparable overall efficacy to fluoride gel in enhancing demineralized primary tooth enamel microhardness despite showing different patterns of demineralization and recovery. These findings suggest that broccoli extract may be a natural and effective alternative for enhancing enamel remineralization in pediatric caries prevention, warranting further clinical investigation.

## 1 Background

Dental caries is a serious public health issue and the leading cause of oral health burden worldwide, with a prevalence of approximately 60–90 percent in school‐aged children [1]. Despite advancements in preventive and restorative dentistry, the incidence of dental caries remains high, particularly among children [2]. Primary dentition plays a crucial role in maintaining the proper masticatory function, speech development, and esthetics while also serving as a space maintainer for permanent dentition [3]. Therefore, preserving the integrity of primary teeth is essential for the overall well‐being of children [4].

The primary etiological factors contributing to the development of dental caries are cariogenic bacteria, frequent consumption of fermentable carbohydrates, and the susceptibility of tooth enamel [5]. Enamel, the hardest tissue in the human body, is composed of highly mineralized hydroxyapatite crystals [6]. However, despite its strength, enamel is constantly subjected to demineralization processes, which can lead to carious lesions [7].

The microhardness of tooth enamel is critical in determining its resistance to demineralization and the progression of dental caries [8]. Numerous studies have investigated various methods to enhance enamel microhardness, including the use of fluoride, casein phosphopeptide‐amorphous calcium phosphate (CPP‐ACP), and nano‐hydroxyapatite [9, 10, 11]. Although fluoride remains the gold standard for caries prevention, its potential side effects, such as dental fluorosis in children and potential toxicity due to overexposure, have raised public health concerns, particularly in regions with high natural fluoride levels in water supplies. These concerns, coupled with the growing interest in natural and biocompatible alternatives, highlight the importance of identifying effective plant‐based solutions for enhancing enamel remineralization [12, 13].

In recent years, there has been a growing interest in using plant‐derived extracts to prevent and manage oral diseases [14]. Broccoli (*Brassica oleracea* var. italica) is a cruciferous vegetable known for its nutritional value and health benefits [15]. It is rich in vitamins, minerals, and bioactive compounds, such as glucosinolates, phenolic compounds, and high dietary fibers [16]. Sulforaphane, a naturally occurring isothiocyanate derived from glucoraphanin in broccoli, has been extensively studied for its anticancer, antioxidant, and anti‐inflammatory properties [17, 18, 19]. Additionally, recent studies have suggested that sulforaphane may possess antimicrobial activity against oral pathogens, including *Streptococcus mutans*, a primary causative agent of dental caries [20, 21]. Moreover, broccoli contains essential minerals such as calcium, phosphorus, and magnesium, which are crucial for the mineralization of dental tissues [22]. However, to the best of our knowledge, no study has specifically evaluated the effect of broccoli extract on the microhardness of primary tooth enamel.

Given the potential benefits of broccoli extract and the importance of maintaining the integrity of primary teeth, this in vitro study aimed to evaluate the effect of broccoli extract on the microhardness of primary tooth enamel and to compare the remineralizing potential of broccoli extract with that of a standard fluoride treatment. The findings of this study could provide insights into the potential use of broccoli extract as a natural remineralizing agent for the prevention of dental caries in children.

## 2 Methods

### 2.1 Sample Preparation and Study Design

This in vitro randomized controlled study investigated the remineralizing potential of aqueous broccoli extract on primary tooth enamel, conducted following the Declaration of Helsinki and approved by the Ethics Committee of Shahed University (IR.shahed.REC.1395.38). The study utilized 30 extracted primary second molars collected from the Department of Pediatric Dentistry at Shahed University. All teeth were obtained with informed consent from patients or their guardians for use in research. Teeth were extracted due to trauma, space loss, or planned orthodontic extractions. Only teeth with intact buccal enamel surfaces without caries, abrasions, cracks, or hypocalcification were included in the study. Primary teeth were chosen due to their lower mineral content and higher organic content compared to permanent teeth, making them particularly susceptible to caries and providing a sensitive model for evaluating remineralization strategies [23]. Teeth were screened using a stereomicroscope (EZ4D, Leica, Olympus, Tokyo, Japan) at x10 magnification to exclude those with caries, abrasions, cracks, or hypocalcification on the buccal surface. Selected teeth were stored in a physiological saline solution, refreshed weekly, and sterilized by soaking in a 1% chloramine T solution for 1 week. Following sterilization, teeth were sectioned longitudinally at the central fossa to create uniform sections for treatment using a disc, and the buccal sections were mounted in acrylic self‐cure blocks, ensuring the buccal surface was oriented consistently parallel to the testing surface. The exposed tooth surfaces were cleaned using a prophy brush and a low‐speed handpiece (500–1500 rpm), followed by wet polishing in eight steps using silicon carbide polishers (Matador Wasser fast) with particle sizes ranging from 800 to 3000 grit.

### 2.2 Microhardness Assessment and Demineralization

Initial surface microhardness was measured using a Vickers hardness tester (M‐g503; Shimadzu Corp, Japan) with a dwell time of 15 s and a load of 50 g. Three indentations arranged in a triangular pattern were made on each tooth sample using a pyramidal diamond indenter, and the length of the diagonal left by the indenter was measured using a built‐in scaled microscope.

Demineralization was induced by soaking the samples in 40 mL of Cola (Khoshgovar, Mashhad, Iran) with a pH of 4.5 for 8 min, a duration determined to induce significant demineralization based on preliminary tests showing substantial enamel surface softening within this timeframe, while also minimizing the potential for excessive enamel erosion that could confound microhardness measurements [24, 25]. The Cola was replaced every 2 min to maintain a consistent acidic challenge and minimize the saturation of the solution with dissolved enamel minerals. Samples were then rinsed with distilled water, and post‐demineralization microhardness values were recorded.

### 2.3 Broccoli Extract Preparation

Fresh broccoli florets, stems, and leaves were washed, air‐dried at room temperature for 48 h, and ground into a fine powder using a commercial blender. In a glass bottle, 200 g of broccoli powder was added to 800 mL of distilled water with a pH of 7.0. The bottle was capped tightly, and the contents were shaken every 6 h for 48 h at room temperature to ensure thorough mixing and maximize the extraction of water‐soluble compounds from the broccoli powder. The broccoli infusion was then filtered through Whatman No.1 filter paper to separate the aqueous extract from the vegetable solids. The obtained filtrate considered a 100% concentration broccoli extract, had a pH of 6.8 (measured using a digital pH meter (Hanna Instruments, Model HI991001), and was diluted accordingly for experimental use. The broccoli extract preparation method was adapted from standard procedures for the aqueous extraction of plant materials [26, 27, 28]. It is important to note that while broccoli is rich in bioactive compounds such as sulforaphane and minerals like calcium and phosphorus, we did not quantify the specific concentrations of these components in our prepared extract.

### 2.4 Treatment and Final Microhardness Assessment

The demineralized teeth were randomly assigned into two groups of 15 specimens. Group B was treated with aqueous broccoli extract, and Group F with 1.23% acidulated phosphate fluoride (APF) gel containing 12,300 ppm fluoride ion (pH 3.5) (Sultan Healthcare Inc, Englewood, New Jersey, USA). Treatments were applied for 10 min by immersing each tooth individually in 2 mL of the respective treatment solution within a well of a cell culture plate, ensuring complete coverage of the enamel surface. After treatment, samples were thoroughly cleaned with distilled water, and final microhardness values were recorded.

### 2.5 Statistical Analysis

Data were analyzed using IBM SPSS Statistics for Windows, Version 28.0 (IBM Corp., Armonk, NY, USA). The normality of the data was assessed using the Shapiro–Wilk test, which indicated that all microhardness measurements followed a normal distribution (*p* > 0.05). Descriptive statistics were presented as mean ± standard deviation (SD) with accompanying 95% confidence intervals (CIs) for continuous variables.

Independent *t*‐tests were used to compare microhardness values between groups at each time point. A two‐way repeated measures analysis of variance (RM‐ANOVA) with Greenhouse‐Geisser correction was performed to analyze changes in microhardness across time points and between groups (broccoli extract vs. fluoride gel). Percentage changes between time points (T1, T2, T3) were calculated and compared between groups using independent t‐tests. All statistical tests were two‐tailed, and a *p*‐value < 0.05 was considered statistically significant.

## 3 Results

The microhardness values were measured at baseline (T1), after demineralization (T2), and after treatment (T3). Table 1 presents the mean, standard deviation, and 95% confidence intervals of Vickers microhardness values for both groups at each time point.

**Table 1:** Mean microhardness (±SD) and 95% confidence intervals of enamel samples in broccoli extract and fluoride gel groups at three time points.

Time point	Broccoli extract (Mean ± SD) [95% CI]	Fluoride gel (Mean ± SD) [95% CI]
**Baseline (T1)**	508.47 ± 49.03 [481.31, 535.62]	464.40 ± 77.83 [421.30, 507.50]
**After demineralization (T2)**	266.73 ± 52.49 [237.67, 295.80]	322.27 ± 35.07 [302.84, 341.69]
**After treatment (T3)**	374.40 ± 35.22 [354.90, 393.90]	386.47 ± 35.29 [366.92, 406.01]

The Independent‐sample t‐test revealed no statistically significant difference between the groups at baseline (*t* = 1.85, *df* = 28, *p* = 0.074). Repeated measures ANOVA revealed a significant interaction between time and group (F(1.43, 40.29) = 6.56, *p* =  0.007), suggesting that the pattern of microhardness changes over time differed between the broccoli extract and fluoride gel groups. This means that assessing the main effects of time and group can be misleading. Although there was no statistically significant difference between the two groups at baseline, the mean difference between the two groups at this time was 44 units. In addition, the interaction between time and group was significant. Therefore, comparing the two groups was based on the percentage of hardness changes. For this purpose, three percent changes were calculated, including (T1 to T2), (T1 to T3), and (T2 to T3). Table 2 presents statistical indices of the changes along with p‐values from the independent‐samples t‐test for between‐group comparisons of the changes.

**Table 2:** Descriptive indices of percentage changes in microhardness between time points with 95% confidence intervals for both groups.

Change period	Broccoli extract (Mean ± SD) [95% CI]	Fluoride gel (Mean ± SD) [95% CI]	*p*‐value^a^
**Demineralization (T1 to T2)**	−47.00 ± 11.82% [40.45, 53.54]	−28.04 ± 18.27% [17.92, 38.16]	0.002*
**Recovery (T2 to T3)**	+44.95 ± 30.00% [28.34, 61.56]	+20.78 ± 12.93% [13.62, 27.94]	0.008*
**Overall change (T1 to T3)**	−25.25 ± 13.60% [17.72, 32.79]	−13.87 ± 19.81% [2.89, 24.84]	0.077

* Statistically significant (*p* < 0.05).

^a^ Based on the independent‐samples *t*‐test.

**Figure 1:**
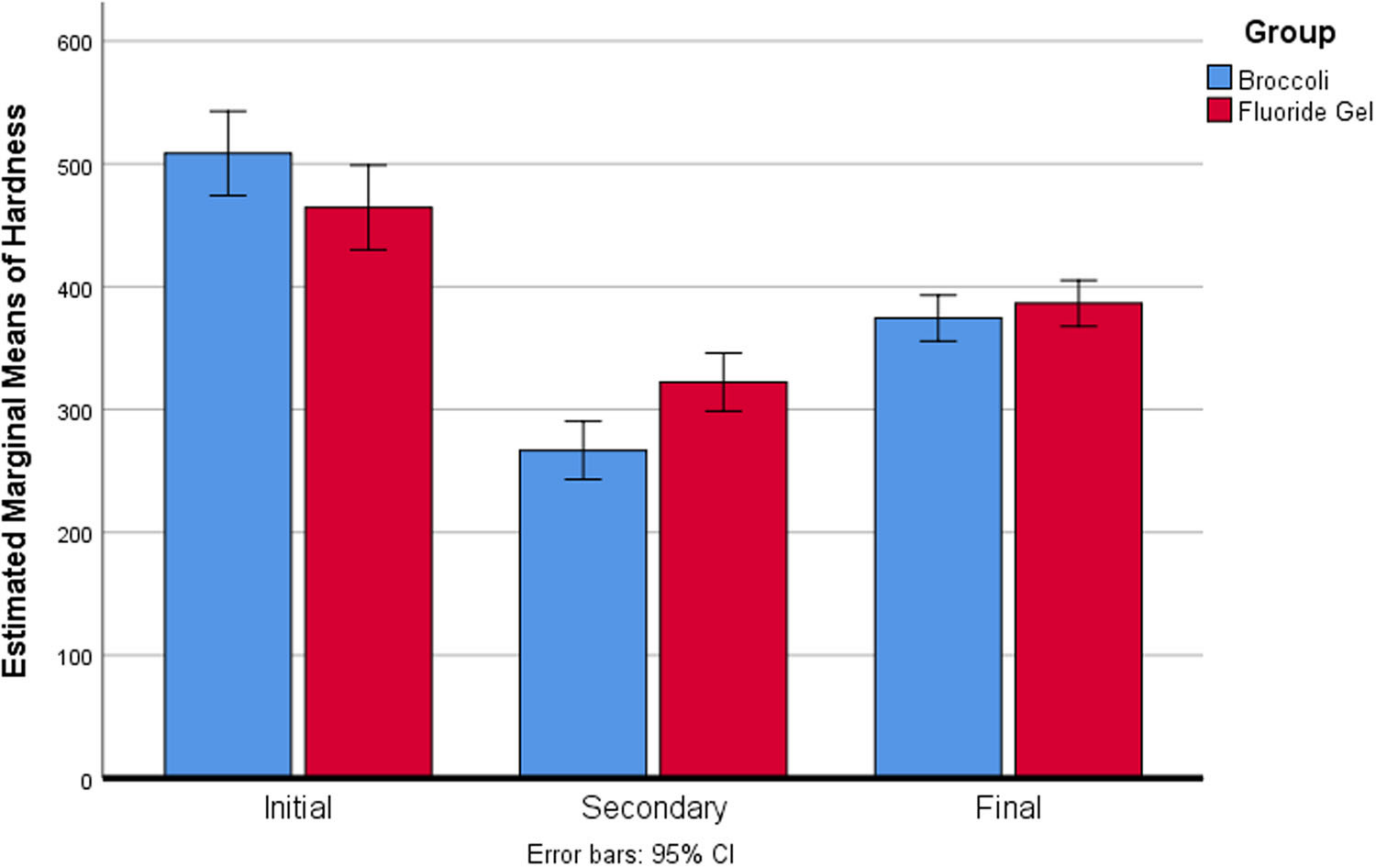
illustrates the changes in enamel microhardness over time for both groups. The broccoli extract group showed a larger decrease in microhardness after demineralization but also a more pronounced increase after treatment.

The broccoli extract group showed a significantly greater initial demineralization (−47.00% vs −28.04%, *p* = 0.002, mean difference = 18.96%) but also demonstrated significantly higher recovery after treatment (+44.95% vs +20.78%, *p* = 0.008, mean difference = 24.17%) compared to the fluoride gel group. The overall change from baseline to final measurement showed no statistically significant difference between groups (*p* = 0.077, mean difference = 11.39%), though the broccoli extract group showed a numerically greater total reduction in microhardness (−25.25% vs −13.87%). Figure 1 illustrates the changes in microhardness at each time point for both groups. The broccoli extract group demonstrated a more pronounced recovery in enamel microhardness compared to the fluoride group.

## 4 Discussion

The present study aimed to evaluate the remineralizing potential of aqueous broccoli extract on artificially demineralized primary tooth enamel and compare its efficacy to fluoride gel, a standard treatment. Our findings revealed that both treatment groups significantly increased enamel microhardness following demineralization. However, the broccoli extract group demonstrated a significantly greater recovery in enamel hardness after demineralization compared to the fluoride gel group.

The finding that fluoride gel effectively increased enamel microhardness aligns with the well‐established role of fluoride in promoting remineralization and enhancing acid resistance of tooth enamel [29]. Fluoride ions interact with the hydroxyapatite crystals of the enamel, forming fluorapatite, which is more resistant to acid dissolution than the original hydroxyapatite [30].

A notable finding was the different patterns of change observed between the two treatments. The broccoli demonstrated a markedly higher recovery rate after treatment (+44.95% vs +20.78%, *p* = 0.008) compared to the fluoride gel group. This distinctive pattern warrants further investigation into the underlying mechanisms.

The superior remineralizing effect of the aqueous broccoli extract compared to the fluoride gel is a novel finding that warrants further discussion. Broccoli is a rich source of various bioactive compounds, including glucosinolates, phenolic compounds, and essential minerals [16]. Sulforaphane, a potent isothiocyanate derived from glucoraphanin in broccoli, has been shown to possess antimicrobial activity against oral pathogens, particularly *Streptococcus mutans* [20, 21]. By inhibiting the growth and adhesion of cariogenic bacteria, sulforaphane may indirectly contribute to the prevention of demineralization and the promotion of remineralization [21]. Furthermore, the phenolic compounds in broccoli have been shown to possess antioxidant properties, which can mitigate oxidative stress in the oral environment [31]. Oxidative stress contributes to enamel demineralization and the progression of dental caries [32]. By reducing oxidative damage, the phenolic compounds in broccoli may help preserve enamel integrity and promote remineralization.

The broccoli's high calcium, phosphorus, and magnesium content may have played a critical role in the observed remineralizing effect [22]. These minerals are essential components of the hydroxyapatite crystals that comprise the tooth enamel [6]. The presence of these minerals in the aqueous broccoli extract could have facilitated the incorporation of calcium and phosphate ions into the demineralized enamel, leading to the formation of new hydroxyapatite crystals and the restoration of enamel microhardness [33].

The pH of the aqueous broccoli extract (6.8) may have also contributed to its remineralizing potential. A slightly acidic pH can enhance the solubility of calcium and phosphate ions, making them more readily available for incorporation into the enamel [34]. Additionally, the near‐neutral pH of the broccoli extract may have minimized the risk of further enamel erosion during the treatment process, a concern associated with the acidic pH of the fluoride gel [35].

The demonstrated efficacy of the aqueous broccoli extract in remineralizing primary enamel suggests that it could be a promising natural alternative for caries prevention in children. This is especially important considering the growing concern about the potential adverse effects of excessive fluoride exposure, such as dental fluorosis (Abanto Alvarez, Rezende, Marocho, Alves, Celiberti, & Ciamponi, 2009). The findings of this study open new avenues for the development of natural, plant‐based remineralizing agents for caries prevention. Incorporating broccoli extract into oral care products, such as toothpaste, mouthwashes, and topical gels, could provide a safe and effective alternative to conventional fluoride‐based treatments.

This study has several limitations. Being an in vitro study, the results may not fully translate to the complex oral environment, which is influenced by factors such as saliva, dietary habits, and oral microbiota. The study focused exclusively on enamel microhardness, an important but limited indicator of enamel health, as it does not account for surface morphology, roughness, or chemical composition such as the calcium and phosphate content of the enamel before and after treatment. Additionally, we did not quantify the specific concentrations of bioactive compounds and minerals in the broccoli extract, which limits its standardization for potential clinical use. Variability in bioactive compound concentrations due to differences in broccoli variety, cultivation conditions, and extraction methods also limits reproducibility. Future research should investigate dose‐dependent effects, optimize treatment durations, and validate these findings in clinical trials to establish the feasibility of broccoli extract as a remineralizing agent for pediatric dentistry.

## 5 Conclusion

This in vitro study provides evidence that aqueous broccoli extract significantly enhances the recovery of enamel microhardness following demineralization, demonstrating a superior recovery compared to fluoride gel. However, the overall change in microhardness from baseline to posttreatment did not differ significantly between the two groups. These findings highlight the potential of broccoli extract as a natural alternative for caries prevention in children, offering a safer and biocompatible option compared to conventional fluoride treatments. Further clinical research is needed to validate these findings under real‐world conditions and to explore the long‐term benefits and safety of broccoli extract in pediatric caries prevention.

## Author Contributions

**Sadighe Mozaffar:** conceptualization, validation, methodology, data curation, visualization, writing – review and editing, writing – original draft, project administration. **Mehrdad Karimi:** supervision, investigation, conceptualization, data curation, validation, methodology, writing – review and editing, formal analysis, resources. **Ali Ismail:** conceptualization, methodology, validation, investigation, writing – original draft, writing – review and editing. **Morteza Banakar:** conceptualization, methodology, data curation, writing – review and editing, writing – original draft, visualization, investigation.

## Ethics Statement

The study was conducted in accordance with the Helsinki Declaration of 1964 and its later amendments. The study was approved by the ethical committee of Shahed University (IR.shahed.REC.1395.38).

## Conflicts of Interest

The authors declare no conflicts of interest.

## Transparency statement

The lead author Morteza Banakar affirms that this manuscript is an honest, accurate, and transparent account of the study being reported; that no important aspects of the study have been omitted; and that any discrepancies from the study as planned (and, if relevant, registered) have been explained.

## Data Availability

The data that support the findings of this study are available from the corresponding author upon reasonable request. The data set includes raw microhardness measurements, statistical analyses, and experimental protocols. All authors have read and approved the final version of the manuscript. Morteza Banakar had full access to all of the data in this study and took complete responsibility for the integrity of the data and the accuracy of the data analysis.
